# CD8^+^ T cell clonotypes from prior SARS-CoV-2 infection predominate during the cellular immune response to mRNA vaccination

**DOI:** 10.21203/rs.3.rs-2146712/v1

**Published:** 2022-10-10

**Authors:** Emily S. Ford, Koshlan Mayer-Blackwell, Lichen Jing, Anton M. Sholukh, Russell St. Germain, Emily L. Bossard, Hong Xie, Thomas H. Pulliam, Saumya Jani, Stacy Selke, Carlissa J. Burrow, Christopher L. McClurkan, Anna Wald, Michael R. Holbrook, Brett Eaton, Elizabeth Eudy, Michael Murphy, Elena Postnikova, Harlan S. Robins, Rebecca Elyanow, Rachel M. Gittelman, Matyas Ecsedi, Elise Wilcox, Aude G. Chapuis, Andrew Fiore-Gartland, David M. Koelle

**Keywords:** SARS-CoV-2, T cell receptor, mRNA vaccine, immune repertoire, clustering analysis

## Abstract

Almost three years into the SARS-CoV-2 pandemic, hybrid immunity is highly prevalent worldwide and more protective than vaccination or prior infection alone. Given emerging resistance of variant strains to neutralizing antibodies (nAb), it is likely that T cells contribute to this protection. To understand how sequential SARS-CoV-2 infection and mRNA-vectored SARS-CoV-2 spike (S) vaccines affect T cell clonotype-level expansion kinetics, we identified and cross-referenced TCR sequences from thousands of S-reactive single cells against deeply sequenced peripheral blood TCR repertoires longitudinally collected from persons during COVID-19 convalescence through booster vaccination. Successive vaccinations recalled memory T cells and elicited antigen-specific T cell clonotypes not detected after infection. Vaccine-related recruitment of novel clonotypes and the expansion of S-specific clones were most strongly observed for CD8^+^ T cells. Severe COVID-19 illness was associated with a more diverse CD4^+^ T cell response to SARS-CoV-2 both prior to and after mRNA vaccination, suggesting imprinting of CD4^+^ T cells by severe infection. TCR sequence similarity search algorithms revealed myriad public TCR clusters correlating with human leukocyte antigen (HLA) alleles. Selected TCRs from distinct clusters functionally recognized S in the predicted HLA context, with fine viral peptide requirements differing between TCRs. Most subjects tested had S-specific T cells in the nasal mucosa after a 3rd mRNA vaccine dose. The blood and nasal T cell responses to vaccination revealed by clonal tracking were more heterogeneous than nAb boosts. Analysis of bulk and single cell TCR sequences reveals T cell kinetics and diversity at the clonotype level, without requiring prior knowledge of T cell epitopes or HLA restriction, providing a roadmap for rapid assessment of T cell responses to emerging pathogens.

## Main

Hybrid immunity - the product of natural infection and vaccination - is more protective against SARS-CoV-2 infection than either infection or vaccination alone^1–3^. Hybrid immunity remains protective against severe disease despite antigenic escape from neutralizing antibodies (nAb), as has occurred with the emergence of variants of concern (VOC)^4^. T cells likely contribute to this protection. Data suggest that in most persons, the recognition of multiple T cell epitopes^5,6^ largely preserves T-cell responses despite VOC evolution^7^, although T cell escape has been observed in a minority of persons^8^. Animal models suggest that T cells found in the airway protect against respiratory coronavirus challenge^9,10^, and recent functional data in humans document tissue-resident memory S-specific T cells after SARS-CoV-2 mRNA vaccination^11^ or breakthrough infection^12^. These data motivated detailed studies of T cells in hybrid immunity.

SARS-CoV-2 infection increases the abundance of memory virus-specific T cells^12–15^. In each person, a diverse repertoire of heterodimeric TCRs recognize SARS-CoV-2 by binding complexes of antigen-derived peptides presented by polymorphic major histocompatibility complex proteins, encoded in humans by human leukocyte antigen (HLA) genes^16^. Clonal selection theory posits that naive T cell clonotypes proliferate in response to antigen exposure and persist as memory^17,18^. Thus, we hypothesized that in the context of hybrid immunity, repeated S antigen exposure from mRNA vaccination after COVID-19 would expand S-specific T cell clonotypes from prior infection and potentially diversify S-specific T cell memory^19,20^.

To investigate diversity and kinetics of S-specific T cells, we used activation-induced markers (AIM) and sequenced the TCRs of antigen-specific T cells (AIM-scTCRseq)^20^. With TCR sequences as molecular barcodes, antigen-reactive cells were quantified longitudinally in immune cell repertoires obtained via high-throughput (i.e., bulk) sequencing of the TRB locus from blood. Matching AIM-scTCRseq with longitudinal bulk repertoire data permitted measurement of the recruitment and expansion of S-reactive clonotypes from convalescence through three mRNA vaccinations to measure clonotype-level kinetics and to evaluate the diversity of S-specific CD8^+^ and CD4^+^ T cells. In the full cohort we sequenced 259 serial bulk *TRB* repertoires from 54 persons developing hybrid immunity with complementary clinical and serologic data. We also performed bulk repertoire sequencing at the infection-relevant nasal site. This permitted examination of the kinetics of S-specific T cell expansion as well as correlations between the severity of COVID-19 illness, the antibody response to vaccination, the longitudinal dynamics of S-specific T cells over time, and the presence of S-specific T cells at the site of pathogen entry. Finally, thousands of TCR sequences were clustered by an HLA-informed sequence similarity algorithm to permit inference of the likely restricting HLA allele for many S-reactive TCRs - providing a broadly applicable roadmap for “de-orphaning” TCR sequences without *a priori* knowledge of epitope-level specificity.

## Results

### Cohort and TRB repertoire sequencing

**Figure 1:**
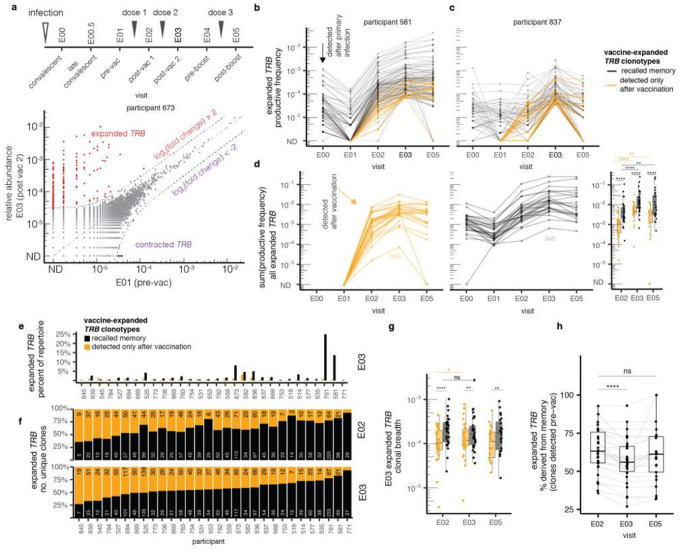
Vaccine broadens pre-existing memory response by expanding low abundance clonotypes. We identified expanded and contracted clonotypes in bulk *TRB* repertoires. Expanded clonotypes are those with log_2_(fold change) > 2 and Fisher’s exact test FDR-adjusted p value < 0.05 when comparing E01 (pre-vaccine) and E03 (post-2nd mRNA vaccine dose) as shown for a representative participant **(a)**. Expanded clonotypes were assigned as detected prior to vaccination (black) versus only detected after vaccination (orange), shown for two representative participants **(b, c)**. For each participant with paired E01 and E03 specimens we computed the sum of productive frequencies of all expanded clonotypes by whether they were previously detected. Comparisons of the relative frequencies of previously seen and unseen clonotypes at each post-vaccine time point are shown in the bar graph at the right (**d**). In the full cohort with E01-E03 matched samples, the summed frequencies of memory and previously unseen vaccine-expanded clonotypes (black) vary between participants after the second vaccine dose. Recalled memory clonotypes (black) predominate. Of note, participant P845, who did not seroconvert after reported natural infection, had the lowest integrated abundance of expanded clonotypes after vaccine dose 2 **(e)**. The relative contributions of memory and previously unseen clonotypes to the diversity of expanded clonotypes varied across the cohort at both E02 and E03 timepoints, with serologically naive participant P845 having the lowest contribution of memory clonotypes. Numbers of unique expanded clonotypes after the 1 st and 2nd mRNA vaccine doses are shown by participant with numbers of unique memory (black) or previously unseen (orange) clonotypes **(f)**. The cumulative frequencies of both memory and previously unseen expanded clonotypes increase after the 2nd mRNA dose compared to the first, but not after a booster. At each timepoint, memory clonotypes from prior infection(s) predominate **(g)**. The proportion of unique expanded clonotypes accounted for by previously detectable *TRB* clonotypes (% memory) decreased during the course of the primary vaccination (**h**). Statistical comparison between paired samples are signed-rank tests, and comparison between groups are Wilcoxon tests. Asterisks represent level of statistical significance (ns not significant, *<0.05, **<0.01, ***<0.001, ****<0.0001).

Bulk TCR beta *(TRB)* repertoires from 259 peripheral blood mononuclear cells (PBMC) specimens - spanning convalescence through booster vaccination - were sequenced from 54 persons reporting PCR-documented COVID-19 between April-August 2020, when the SARS-CoV-2 ancestral-like D614G strain prevailed^21^. The cohort has been described^22–25^. The 18 hospitalized and 36 non-hospitalized SARS-CoV-2-infected persons had serial blood sampling through mRNA vaccination (demographics in **Supplemental Data Table 1**). Infection status in one hospitalized participant (P845) was ambiguous due to undetectable anti-nucleocapsid and anti-spike serum antibody before vaccination. The earliest blood *TRB* repertoires (collected at visit E00) (timeline, Fig. 1a; per-participant timelines, **Extended Data Fig. 1**) were obtained a median of 78.5 (IQR 56–105) days after COVID-19 diagnosis. A pre-vaccine *TRB* repertoire (at visit E01) was obtained from 34 participants immediately before vaccination, a median of 369 days (IQR 333–390) after symptom onset. Most participants had a *TRB* repertoire 2.5 to 4 weeks after mRNA dose 1 and prior to dose 2 at visit E02 (N = 52, median 19.5 days post dose 1, IQR 15–24) and after dose 2 at E03 (N = 53, median 24 days post dose 2, IQR 20–28). Forty-four persons had an mRNA booster (third dose) a median of 259 (IQR 230–283) days after primary vaccination, and a *TRB* repertoire at E05 (median 41.5 days after booster, IQR 26–69). Three participants (P545, P664, and P669) had increased anti-SAR-CoV-2 nucleoprotein (N) plasma IgG between E03 and E05 visits, representing breakthrough reinfection after primary vaccination (**Extended Data Fig. 1, Supplemental Data Fig. 1, Supplemental Data Table 2**). Data from after primary vaccination from these participants are excluded from statistical analyses. Blood *TRB* sequencing recovered medians of 429,661 productive *TRB* templates and 242,861 unique productive *TRB* rearrangements (‘clonotypes’) per sample (**Supplemental Data Table 3**).

### Vaccination provokes clonal expansion

To assess the impact of vaccination on repertoire structure, we compared *TRB* clonotype frequencies within-participant across time by pairwise differential relative abundance. Longitudinal repertoire sampling was used to measure clonal expansions and contractions following vaccination (representative participant, Fig. 1a; persons with E01 and E03 samples, **Extended Data Fig. 2**). Some participants had large expansions of individual *TRB* clonotypes - up to 1,000-fold or greater - after primary vaccination. To differentiate changes in clonal frequency likely due to vaccine-induced proliferation from natural background variation, we defined expanded and contracted clones as those with an absolute log_2_ fold- change greater than 2 and a statistically significant change in frequency between samples using Fisher’s Exact Test with correction for multiple hypotheses (FDR-adjusted q-value < 0.05) (**Supplemental Data Table 4**, summary in **Supplemental Data Table 5**). This focuses the analysis on clonotypes with a robust and statistically significant expansion or contraction.

Among 32 participants with *TRB* repertoires at both E01 and E03, a median of 72 (IQR 51–104) statistically significant vaccine-expanded *TRB* clonotypes were detected per participant, comprising a median of 1.8% (IQR 0.9–3.5%) of each participants’ productive circulating T cell repertoire. In one extreme case, 292 unique vaccine-expanded *TRB* clonotypes made up more than a quarter of the circulating repertoire after primary vaccination in participant P761 (**Extended Data Fig. 2**). We noted pronounced intra-cohort and dose-to-dose variability in the number and fold-expansion of expanding clonotypes as well as their pre-vaccine frequencies (two representative participants, Fig. 1b, c).

To track vaccine-expanded clonotypes over time, the productive frequencies of clonotypes meeting criteria for expansion between E01 and E03 were plotted over the time course from convalescence through booster for each participant (**Extended Data Fig. 3a**). Two representative participants illustrate heterogeneity in the vaccine T cell response. In participant P581 (Fig. 1b), many *TRB* clonotypes destined for vaccine-related expansion were present at 0.001 to 0.1 % of the repertoire at early COVID-19 convalescence (E00). These clonotypes universally decreased in frequency between infection and vaccination. The rarer convalescent clonotypes tended to contract below the limit of detection (ND in Fig. 1b) but then proliferated at least 10- to 100-fold after the first mRNA dose (black lines in Fig. 1b between E01 and E02). In participant P581, there were also previously unseen clonotypes, observed in neither convalescent (E00) nor pre-vaccination (E01) repertoires, that expanded after dose 1 (shown as orange line in Fig. 1b The 2nd dose provoked only modest additional proliferation for clonotypes that expanded after the 1st dose and entrained almost no additional clonotypes (Fig. 1b). In P581, 80% of the vaccine-expanding clonotypes were detected before vaccination, suggesting a dominant role for memory T cells. Dose 2 contributed few additional vaccine-expanded clonotypes and there was sparse evidence of clonotype expansion after a third dose (E05, Fig. 1b). In contrast, for participant P837 (Fig. 1c), clonotypes destined to expand after vaccination displayed much more kinetic heterogeneity, with clonotypes detected at E00 showing either stability or contraction prior to vaccination. Dose 1 prompted expansion in many previously unseen clonotypes but did little to expand the clonotypes persisting prior to vaccination. Dose 2, however, provoked expansion of previously-detected clonotypes, as well as entrained additional new clonotypes not observed in earlier repertoires. In the year after the primary vaccine series, the clonotypes that had required two doses to expand tended to contract markedly, despite receipt of a 3rd dose (E05, Fig. 1c). Clonotype trajectories for the remaining 30 participants with E01 and E03 samples (**Extended Data Fig. 3a**) further illustrate inter-participant variability. While the majority of both memory and previously undetected vaccine-expanded clonotypes persisted throughout the time course, a higher percent of recalled memory clonotypes persisted past the 3rd dose to E05 (p-value 0.002, **Extended Data Fig. 3b**).

### Vaccination boosts pre-existing clonotypes and elicits previously undetected clonotypes

The vaccine-responsive clonotypes detected in blood before vaccination likely represent memory to SARS-CoV-2 or other CoV infection(s), while the previously unseen vaccine-responsive clonotypes could be immunologically naive or rare memory clonotypes below the limit of detection. With a median repertoire size of 430,000 sequenced productive templates, a clonotype with a frequency of 1 cell per 200,000 would have > 85% chance of detection. While we could not differentiate these possibilities, we summarized recalled memory and previously unseen clonotypes over time across our cohort to compare the kinetics of these subpopulations and their relative contributions to the post-vaccine T cell response. After dose 1 (E02), the integrated abundance of previously unseen clonotypes comprised 0.01–1% of the total repertoire (Fig. 1d), whilst memory clonotypes contributed 0.1 *%* to almost 10% of the repertoire. To assess cellular proliferation after each vaccine dose, we examined clones present at the pre-vaccine timepoint (E01) that expanded by E03. For these clonotypes, the median number of cell divisions after dose 1 (4.0 [2.4, 4.6]) was greater (p-value < 0.001) than after dose 2 (1.8 [1.2, 2.7]). By E03, after two mRNA doses, total frequencies of expanded clonotypes ranged considerably from 1 to 26% (Fig. 1e). T cells derived from memory were numerically dominant (Fig. 1e), but the diversity of unique clonotypes was more balanced between memory and previously unseen vaccine-responsive clonotypes (Fig. 1f).

To further analyze heterogeneity in vaccine response among all participants we calculated the (i) frequency and (ii) clonal breadth quantities within each repertoire from the defined set of expanded clonotypes. Frequency is the sum of the productive frequencies of all expanded clonotypes in a sample, whereas clonal breadth is the proportion of unique clonotypes that were expanded, an indicator of clonal diversity. We observed significant increases in the frequency of expanded clonotypes between visit E02 and visit E03 for both recalled memory and previously unseen clonotypes (Fig. 1d, right bar graph, p < 0.001). The breadth of previously unseen clonotypes also increased significantly after each dose (Fig. 1g, p < 0.05). The percentage of unique *TRB* clonotypes derived from memory T cells decreased from visit E02 to visit E03 in most participants (Fig. 1h, p < 0.001 signed-rank test), indicating that both doses expand naive or rare memory lymphocytes. Expanded *TRB* clonotypes that were detectable only after vaccination often persisted after the booster (**Extended Data Fig. 3**) but were less abundant than expanded memory clones (Fig. 1d, right bar graph). Notably, the repertoire of each person with a frequency of vaccine-expanded clones > 5% at E03 (participants P673, P582, P836, P761, and P581) was characterized by strong polyclonal expansion of pre-existing cellular memory (Fig. 1e, f).

### Single cell sequencing identifies CD8 ^+^ phenotype in highly expanded clones

To obtain paired TCRαβ sequences and determine the phenotypes of vaccine-expanded clonotypes, we isolated S-reactive T cells. We chose the post-2nd dose sample (E03) from 17 participants with > 50 vaccine-expanded clonotypes (Supplemental Data Table 5). The subgroup was balanced for sex and vaccine manufacturer; four were hospitalized with COVID-19. T cells co-expressing the activation-induced markers (AIM) CD69 and CD137 after S peptide stimulation were single-cell sequenced (AIM-scTCRseq) at *TRA* and *TRB* loci. Clonotypes were classified as S-reactive if their productive *TRB* frequency was enriched within the AIM-scTCRseq fraction relative to their *TRB* bulk unsorted frequency at the same time point. From 17 E03 samples, we sequenced and phenotyped 24,557 S-reactive T cells and identified 5,733 unique S-reactive clonotypes (see Methods) (**Supplemental Data Tables 6,7**). S-reactive clonotypes were individually classified as CD4^+^ or CD8^+^, or inconclusive, using oligonucleotide-labeled mAb binding (**Supplemental Data Table 7**).

**Figure 2:**
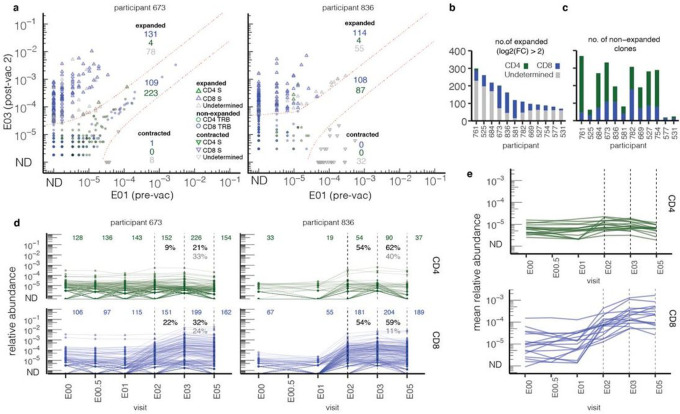
Kinetics of S-reactive clonotypes defined by AIM-scTCRseq spanning convalescence and vaccination. Overlay of *TRB* sequences from AIM-scTCRseq of T cells activated in response to S peptides onto bulk blood *TRB* clonotype frequency comparisons between pre (E01)- and post (E03)-vaccine in representative participants (P836 had no E00.5 sample). The numbers of expanded or contracted *TRB* clonotypes also seen in the AIM-scTCRseq datasets are shown in color while the numbers of such clonotypes not seen by AIM-scTCRseq are gray. Clonotypes are color-coded by CD8+ (blue) or CD4+ (green) phenotypes. Upward and downward triangles indicate expanded and contracted clonotypes. For clonotypes neither expanded nor contracted, only AIM-scTCRseq *TRB*-matched clonotypes are shown (**a**). PBMC *TRB*-defined clonotypes that match AIM-scTCRseq-derived CD8+ (blue) or CD4+ (green) S-reactive *TRB* clonotypes in 12 participants with E01-E03 matched samples are enumerated as the numbers of expanded (**b**) or neither expanded nor contracted clonotypes (**c**). Longitudinal tracking of AIM-scTCRseq identified S-reactive TRB clonotypes shown as relative abundances in PBMC across time in representative participants. Numbers in top rows indicate the number of unique AIM-scTCRseq *TRB* clonotypes, from E03, detected at each time point. Percentages in black refer to the fraction of AIM-scTCRseq clonotypes detected at the E02 and E03 timepoints, respectively, that were not detected at any pre-vaccination time point. Percentages in gray represent the fraction of unique clonotypes detected at E03 that are below the level of detection at E02 (**d**). Mean abundances of CD4 and CD8 S-reactive clonotypes identified by AIM-scTCRseq in 17 participants across time from convalescence through mRNA booster vaccination (**e**).

Mapping S-reactive single-cells to matching *TRB* in serial bulk-sequenced PBMC repertoires revealed substantial overlap (Fig. 2a) with the E03/E01 expanded clonotypes discussed above. In the 12 participants with both AIM-scTCRseq and paired E03/E01 *TRB* bulk data, a median of 34% (IQR 26–53%) of the expanded *TRB* sequences matched a clonotype recovered by AIM-scTCRseq. AIM-scTCRseq recovered almost equal numbers of CD4^+^ and CD8^+^ clonotypes overall (**Supplemental Data Tables 6,7**); however, 93% of the robustly vaccine-expanded S-reactive clonotypes were CD8^+^ (Fig. 2b). Many of these CD8^+^ S-reactive clonotypes were not detected pre-vaccine (ND, Fig. 2a). Most AIM-scTCRseq S-reactive clonotypes that were CD4^+^ did not expand markedly after vaccination (Fig. 2c, **Extended Data Fig. 4**).

Overlay of AIM-scTCRseq data onto serial blood *TRB* repertoires showed that among expanded clonotypes, the frequency in serial blood *TRB* repertoires was highly correlated with the frequency in the AIM-scTCRseq fraction (**Extended Data Fig. 5a**; representative subjects, **Extended Data Fig. 5b, c**). Despite this overall agreement, it was notable that a large fraction of S-reactive clonotypes were not significantly expanded in the bulk *TRB* repertoires, suggesting that not all S-reactive clonotypes were expanded by vaccination and thus would not be identifiable by pre-post *TRB* differential testing alone (Fig. 1). Conversely, there were also some robustly vaccine-expanded *TRB* clonotypes that did not match a corresponding S-reactive AIM-scRNAseq *TRB* (Fig. 2a, **Extended Data Fig. 4, 5**), possibly reflecting vaccine-unrelated changes in the repertoire or suboptimal sensitivity of the AIM-scTCRseq assay. Despite these possibilities, AIM-scTCRseq recovered a balanced spectrum of unique, antigen-reactive CD4^+^ and CD8^+^ T cells across all participants used as a set of index clonotypes to compare the clonal dynamics of S-specific CD4^+^ and CD8^+^ T cells.

### CD4 ^+^ and CD8^+^ S-reactive T cells exhibit divergent kinetics

We matched the S-reactive *TRB* AIM-scTCRseq nucleotide sequences, from E03, to bulk *TRB* repertoires across time (representative participants, Fig. 2d; others, **Extended Data Fig. 6**). Twelve participants had matched *TRB* bulk repertoire sequencing at E00, E01, E02, E03, and E05 time points. Unsupervised clustering of clonotype-level trajectories in these 12 participants revealed that 5 groupings described the expansion or contraction trajectory of most T cell clonotypes (**Extended Data Fig. 7**): (i) minimal proliferation, (ii) proliferation after dose 1 followed by contraction, (iii) late proliferation after dose 2, (iv) proliferation without contraction, or (v) serial proliferation after both dose 1 and 2. CD8^+^ S-reactive T cells were observed to have more prolonged and greater expansion; in 12 of 12 participants CD8^+^ clonotypes were observed to have serial expansion (type v). The majority of CD4^+^ S-reactive T cells showed minimal expansion (type i). CD4^+^ clonotypes were not observed to have strong serial expansion and many contracted after dose 1 or 2 (**Extended Data Fig. 7**). Overall, comparison of the mean trajectories of S-reactive clonotype abundances for the 17 participants with AIM-scTCRseq data (Fig. 2e) illustrates the greater expansion of CD4^+^ than of CD8^+^ T cells after vaccination.

Despite less CD4^+^ clonal expansion in response to vaccination, longitudinal analyses of their S-reactive clones suggest a somewhat stable CD4^+^ S-reactive memory population within the blood. Their integrated abundance, defined as *TRB* sequences in blood matching CD4^+^ AIM-scTCRseq sequences, was 0.06% (IQR 0.02 to 0.1 %) of the circulating repertoire after infection at time point E00, increasing to only 0.09% (IQR 0.04 to 0.2%) after two mRNA doses at E03. The breadth of S-reactive CD4^+^ T cell clonotypes increased from E00 to E02 in 11 of 17 participants (p-value 0.02, signed-rank test). However, the breadth of S-reactive CD4^+^ T cells appears primarily to reflect the recruitment of pre-existing clones; a median of 65% (IQR 61–77%) of S-reactive CD4^+^ clonotypes observed after vaccination (E03) were detected in pre-vaccine samples (**Supplemental Data Table 8 columns X:AE**).

Similar to CD4^+^ cells, diverse S-reactive CD8^+^ *TRB*-defined clonotypes were detected at the visit after infection. Prior to vaccination, 9–14 months after the infection (E01), S-reactive CD8^+^ T cells comprised 0.09% (IQR 0.04–0.2%) of the circulating repertoire, similar in frequency to CD4^+^ T cells at this timepoint (median 0.06%, IQR 0.01–0.1 %, p = 0.09). While some CD8^+^ T cell clonotypes were maintained at detectable levels from early post-convalescence (E00) to pre-vaccination (E01), others contracted below the limit of detection or fluctuated between rare and undetectable. Divergence of persistent versus contracting S-reactive CD8^+^ clonotypes - shown as flat vs. downwardly sloping blue lines from E00 to E01 - is well-visualized in participant P836 (Fig. 2d) and participant P581 (**Extended Data Fig. 6**). Vaccination expanded memory CD8^+^ S-reactive clonotypes to a much greater extent than CD4^+^ S-reactive clonotypes. CD8^+^ S-reactive clonotypes increased in cumulative productive frequency 8-fold to a median of 0.7% (IQR 0.3–0.9%) of participants’ circulating repertoires after the first dose, and further double to 1.5% (IQR 0.8–3%) after the second dose. A similarly high level of S-reactive CD8^+^ clonotypes, 2.3% (IQR 0.6%−4.1 %), remained after a third vaccine dose (p = 0.8). In comparison, after the second dose, S-reactive CD4^+^ T cells increased in productive frequency by slightly under 2-fold, to 0.09% (IQR 0.04–0.2%), significantly less than CD8^+^ T cells (p < 0.0001). Also, unlike CD8^+^ T cells, the productive frequency of S- reactive CD4^+^ T cells declined slightly from after dose 2 to after the booster dose, to 0.085% (p = 0.002 signed-rank test) (Fig. 2d).

The data illustrate that repeated antigen exposure likely diversifies detectable S-reactive CD8^+^ T cells; a median of 50% (IQR 33–61%) of the CD8^+^ *TRB* clonotypes observed after the second mRNA dose were not detectable prior to vaccination. Furthermore, a median 20% (IQR 11–32%) of the S-reactive CD8^+^ *TRB* clonotypes observed after dose 2 were also not yet detectable after dose 1. Thus, even in persons with a pre-existing S-reactive memory T cell population due to previous infection, a second mRNA dose may recruit previously naive or memory CD8^+^ T cells clonotypes, potentially capable of broadening recognition of S. The additional boosting effect of a two-dose vaccine regimen can be seen visually by longitudinal clonal tracking between E02 and E03 for participant P836 (Fig. 2d), and in participants P684, P527, and P525 (**Extended Data Fig. 6**).

To study the potential emplacement of SARS-CoV-2-specific T cells in the nose, a site of viral infection, we obtained nasal swabs at E05, several weeks after booster vaccination, from 48 persons. Productive *TRB* sequences were obtained from between 184 to over 57,000 T cells per swab. Amongst 16 persons with nasal samples and AIM-scTCRseq data, 14 had confirmed S-reactive CD8 T cell clonotypes detected in nasal swabs. Considerable heterogeneity was noted, as exemplified by recovery of diverse and abundant S-specific CD8 T cells in participant P673, but only a few clonotypes and at low abundance were detected in a nasal swab participant P836. S-specific CD4^+^ T cells were occasionally also detected (**Extended Data Fig. 9**).

### Hybrid immunity elicits highly public S-specific TCR motifs

**Figure 3:**
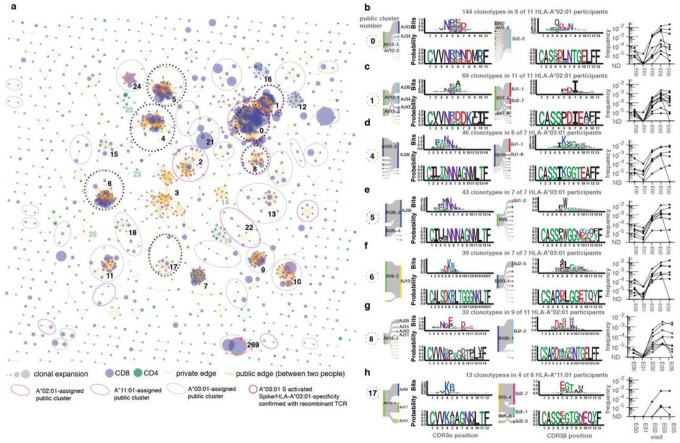
Sequence similarity network shows public CD8 responses among TCRαβ sequences recovered by AIM-scTCRseq. Sequence similarity graph with 1448 clonotypes and 248 convergent CD8 (blue) and CD4 (green) clusters of two or more S-reactive clonotypes recovered from 17 individuals following second mRNA immunization (**a**). Representative logo plots for cluster numbers indicated with integers in (**a**) with inferred restricting HLA class I alleles (**b-h**). The same TCRαβ AA sequences could be included more than once if they were found in distinct participants. In (**a**), edges are formed between similar receptors (TCRdist ≤ 100). Yellow edges indicate public connections between similar TCRs from distinct participants. Dashed edges connect clonotypes found in a single participant. The size of circles represent the degree of clonal expansion as estimated by the number of single-cell droplets within which each unique TRA/TRB clonotype was observed. The HLA allele most statistically associated with each cluster (see Methods) is shown to the upper left of each cluster diagram (**b-h**). For each CDR3 motif, the lower sequence logo shows the probability of each amino acid residue at each CDR3 position and the upper sequence logo depicts the information content in bits comparing the residue usage to a set of randomly selected CDR3 with the same V and J gene usage frequencies as observed in the sequence cluster (see Methods). Residues are colored by chemistry, acidic (red), basic (blue), hydrophobic (black), neutral (purple), polar (green). Line graphs at right are time courses of the sum of the abundances of *TRB* sequences from whole blood repertoires contained within the indicated TCR cluster, with each line representing the productive frequency of clonotypes within that cluster for an individual participant with the indicated HLA allele.

TCRs recognizing a common ligand often exhibit convergent sequence features^26,27^ in CDR3 peptide-contacting residues and other CDRs that contact the peptide-HLA ligand. Amongst 5,733 unique S-reactive *TRA/TRB* clonotypes recovered by AIM-scTCRseq from 17 participants and passing additional quality filters (see Methods), we computed pairwise sequence divergence using TCRdist - a multi-CDR position-weighted, biochemically aware distance metric - to search for public TCR clusters with similar CDR AA sequences^28^. A similarity graph was constructed from the 1,458 clonotypes that had at least one other similar TCR in the dataset, with edges joining sufficiently similar TCRs (TCRdist metric ≤ 100, generally corresponding to similar TRBV/TRAV gene usage and one to four AA substitutions or deletions within TRA and TRB CDR3s). This graph of high-confidence SARS-CoV-2 S-specific TCRαβ sequences, identified without prior knowledge of the restricting HLA or peptide, enabled us to reveal diverse public clusters of TCRs, often characterized by distinct CDR3 motifs, that were expanded by mRNA vaccination. Overall, we found 284 such TCR clusters (Fig. 3a, **Extended Data Figs. 8, 9**). The ten largest clusters contained cells from 3–11 participants and contained between 25–144 unique member clonotypes.

We reasoned that if public clusters accurately identified groups of TCRs recognizing a shared epitope, each TCR group should contain exclusively CD8^+^ or CD4^+^ T cells and be enriched for participants sharing at least one HLA allele. Consistent with this hypothesis, greater than 97% of edges within clusters connected clonotypes with matching CD4^+^ or CD8^+^ assignments (Fig. 3a). Many of the public TCR clusters were formed from groups of persons expressing only one feasible shared HLA class I or class II allele, suggesting specificity for a peptide ligand restricted by this allele (**Extended Data Fig. 9**).

Cluster analyses revealed both previously identified and novel public TCRαβ paired motifs, containing clonotypes expanded by vaccination, most notably in persons expressing the HLA-A*02:01, A*03:01, or A*11:01 alleles (Fig. 3a, **Extended Data Figs. 8, 9, Supplemental Data Fig. 2**). The two largest public receptor clusters (Cluster 0 and Cluster 1) correspond with previously identified NTGEL-TRBJ2–2 (Fig. 3b) and PDIE (Fig. 3c) motifs recognizing the HLA-A*02:01-restricted epitope YLQPRTFLL (S amino acids 269–277, S_269–277_)^7,20,29–32^. Interestingly, while receptors from these two large public clusters were ubiquitous among A*02:01-expressing persons prior to and after vaccination, a related HLA-A*02:01-assigned motif (Cluster 8, Fig. 3g) was only commonly observed after vaccination. The receptors forming Cluster 8 were distinguished from Cluster 0 receptors by longer 14 AA CDR3α and 16 AA CDR3β lengths and strict *TRAV12–1* and *TRBV29–1* usage. Notably, these receptors have a much lower probability of generation (median Pgen CDR3α = 5.1×10^−9^, CDR3β = 9.7×10^−11^) versus those in Cluster 0 (median Pgen CDR3α = 2.2×10^−8^, CDR3β = 1.5×10^−8^)^18^, yet Cluster 8 TCRs were consistently expanded after exposure to the vaccine in 8 of 11 HLA-A*02 participants, suggesting that vaccination may diversify the set of circulating receptors to include more variable length cognate receptors targeting immunodominant epitopes.

We also observed multiple novel and highly public HLA-A*03:01-associated clusters - with sequence motifs found in at least 6 of the 7 A*03:01-expressing participants. Two of largest HLA-A*03-associated clusters shared CDR3α junctions defined by central NNNAG residues, which were paired with two distinct and V-gene biased CDR3β receptor motifs in Cluster 4 (TRBV19-dominated) and Cluster 5 (TRBV9-dominated) (Fig. 3d, e). Cluster 4 and 5 motifs shared similar central CDR3β junctional residues (i.e., SIKGG (Fig. 3d) and SPWGG (Fig. 3e)), where a hydrophobic residue in TRB CDR3 position 6 was frequently followed by a glycine residue in positions 7 or 8. This pattern also appeared as a unifying feature within the CDR3β motif of public HLA-A*03:01-associated cluster 6 (Fig. 3f). The prevalent HLA-A*11:01-associated receptor motif, found in 4 of 6 HLA-A*11:01 participants, is shown in Fig. 3h. Visualizations of selected additional TCR clusters with HLA assignments are in **Extended Data Fig. 8**. S-reactive TRA/TRB sequences in public motifs of 3 or more unique sequences are in **Supplemental Data Fig. 2**.

To confirm the ligands of representative TCRs assigned to HLA-A*03:01, we expressed 6 AIM-scTCRseq-identified receptors from participant P673 (Fig. 1a, 2a) that were expanded strongly after vaccination, originating in clusters 5, 6, 10 (two TCRs with identical *TRA)*, 24, and 269 (Fig. 3a). Each TCR showed strong, specific recognition of artificial antigen presenting cells (aAPC) co-expressing HLA-A*03:01 and either full-length S from ancestral strain Wu-1, or near full-length S from Wu-1 or Omicron BA.1, BA2, or BA.4 SARS-CoV-2 (representative data, **Extended Data Fig. 10a-e**; summary, **Extended Data Fig. 10f**). Control APC expressing empty vector, S alone, other HLA-A or B from participant P673 with S, or HLA alone, were negative. All TCRs from showed reactivity with peptide S 378–387, an HLA-A*03:01-restricted epitope^33^ (**Extended Data Fig. 10f,g**). We observed potential differences in the structural requirements for T cell activation between the various TCRs, indicating that the biochemically-informed TCRdist metric may cluster TCRs into functionally meaningful groups (**Extended Data Fig. 10g**). TCRs 1 and 4, from clusters 269 and 6, respectively, recognized 10-mer peptide 378–387 but neither internal 9-mer, requiring both N-terminal lysine 378 and C-terminal leucine 387. In contrast, TCR3 was versatile, equally recognizing the parent 10-mer and each 9-mer. TCR2 was intermediate, optimally recognizing 378–387 with partial response to both internal 9-mers. TCRs 8.1 and 8.2 were generally similar to TCR1/TCR4. In agreement with the transfection data, peptide containing variant amino acids at positions 373, 375, and 376, representing Omicron BA.4/BA.5, was recognized by each TCR. The *TRB* sequence of each reporter cell-confirmed S-specific TCR was detected in the nasal swab of participant P673, some at high abundance (**Extended Data Fig. 9**).

### Severe disease imprints the S-reactive CD4 ^+^ T cell population

AIM-scTCRseq overlay on differential abundance plots (Fig. 2a, **Extended Data Fig. 4, 5**) showed that most highly vaccine-expanded clonotypes were CD8^+^. S-reactive. Expanded CD4^+^ T cells were present, but not as strongly expanded or entrained into the circulating repertoire by vaccination (Fig. 2a, b, e, **Extended Data Fig. 4**). To more generally study the heterogeneity and longitudinal dynamics of SARS-CoV-2 reactive CD4^+^ T cells, we counted the breadth of clones in each sample exactly matching a set of CD4+-associated *TRB* sequences that were previously found to be enriched in SARS-CoV-2 convalescent versus healthy control repertoires^34^ *(diagnostic* breadth). These *TRB* sequences, derived using the ImmunoSEQ assay (Adaptive Biotechnologies)^35–37^, were previously assigned to S (n = 917) or non-Spike (n = 1564) SARS-CoV-2 antigens^38^ (see Methods).

**Figure 4:**
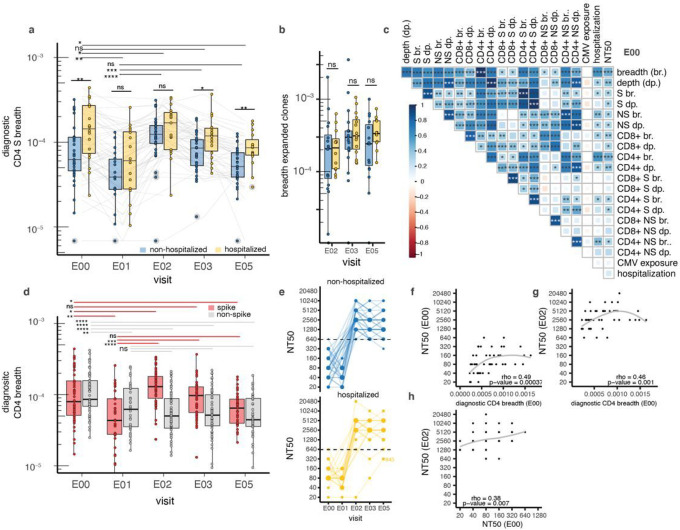
Severity associations between *TRB* sequence-defined metrics and nAb before and after mRNA vaccination. Percentage of the *TRB* repertoire (diagnostic breadth) identified as S-specific CD4+ T cells by *TRB* sequence enrichment in non- vs. post SARS-CoV-2 infection cohorts, distinguished by hospitalization for COVID-19. Boxes represent median and IQR. At top, horizontal lines represent paired Wilcoxon rank sum tests comparing E00 convalescent or E01 pre-vaccination diagnostic breadth with later values. Lines above each time point represent comparisons between participants by disease severity by unpaired Wilcoxon rank sum test **(a)**. The breadth of all expanded clonotypes defined within-participant by comparing E01 and the later specimens indicated on the X-axis did not vary significantly by hospitalization status **(b)** Spearman correlation matrices of *diagnosticsequence-de* fined metrics (S, spike; NS, non-spike, breadth (br.), depth (dp.) for CD4 or CD8-assigned TCRs) with hospitalization and neutralization titers (NT50); all data are from E00. CMV exposure indicates CMV serostatus imputed from *TRB* repertoire. Shading indicates strength of correlation (ρcorrelation coefficient) and asterisks indicate level of statistical significance **(c)**. Breadth of inferred CD4 spike and non-spike-specific *TRB* sequences indicates a waning of breadth over the first year of convalescence, followed by robust increases in spike-specific breadth with vaccination and stability of non-spike-specific breadth. Lines represent paired Wilcoxon signed rank testing between E00 and E01 and later time points **(d)** Neutralization titers (NT50) increased in all participants after the first dose of mRNA vaccine with the exception of participant P845, and remained stably elevated after subsequent doses, regardless of hospitalization status **(e)**. Post-convalescent diagnostic CD4 breadth at E00 was associated with nAb NT50 at E00 **(f)** and E02 **(g)**, there was also an association between E00 and E02 nAb NT50 **(h)** Participant P845 is excluded from correlation analyses and data from E05 in 3 participants experiencing breakthrough infection after 2 mRNA doses is not shown. Level of statistical significance is indicated: ns = not significant, *p<0.05, **p<0.01, ***p<0.001, ****<0.0001.

To examine if COVID-19 disease severity resulted in differential imprinting of the T cell repertoire, we compared *diagnostic* clonal breadth (defined in Methods) in hospitalized and non-hospitalized patients, a measure of antigen-specific diversity computed as the percentage of unique *diagnostic* SARS-CoV-2 reactive *TRB* clonotypes assigned to CD4^+^ T cells amongst the total number of clonotypes detected (Fig. 4a, **Supplemental Data Table 8 columns E:V**). At the convalescent time point (E00), we observed greater *diagnostic* S-reactive CD4^+^ T cell breadth in hospitalized vs. non-hospitalized patients (0.014 vs 0.006%, p-value < 0.01). This difference was no longer observed by the pre-vaccination time point (E01) or after the first mRNA dose (E02). CD4^+^ S breadth was again elevated in previously hospitalized persons after the 2nd (E03) and 3rd (E05) doses (0.012 vs 0.008%, p-value = 0.02 at E03; 0.009% vs 0.005%, p-value < 0.01 at E05), suggesting that a diverse CD4^+^ memory population after severe infection may result in a more diverse repertoire after full vaccination. In contrast, the breadth of vaccine-expanded clonotypes at post-vaccine time points, which was skewed towards CD8^+^ T cells, did not correlate with hospitalization status (Fig. 4b). Spike and non-spike *diagnostic* breadth were weakly correlated (rank correlation ρ = 0.38, p = 0.019) at E00 (Fig. 4c), but the relationship was not evident after vaccination (ρ = 0.14, p = 0.2) (E03, **Supplemental Data Fig. 3**), consistent with increasing *diagnostic* breadth for CD4^+^ recognizing S but not non-S epitopes after mRNA vaccination. Both S and non-S *diagnostic* breadth declined in the year between convalescence and vaccine dose 1 (E00 to E01, p < 0.001). *Diagnostic S* breadth increased promptly after dose 1 and then slowly declined, while in contrast non-S breadth remained stable throughout vaccination after the initial decline in the participants without breakthrough infection (Fig. 4d). We did not observe any associations between CMV infection status and parameters of SARS-CoV-2 specific *TRB* repertoires early after infection (Fig. 4c) or at later time points (**Supplemental Data Fig. 3**).

### Post-infection CD4 ^+^ T cell breadth correlates with antibody responses to vaccination

In the larger cohort that contains the persons studied here, we reported that in the first year after recovery from COVID-19, higher Nt50 was associated with COVID-19 requiring hospitalization^25^. In the subset of subjects reported here, vaccination led to robust increases in Nt50 in previously infected persons, with no difference per hospitalization (Fig. 4e, **Supplemental Data Table 2**). Nt50 declined in the year after infection (reciprocal dilution, median 80, IQR 50–160 at E00 to median 60, IQR 40–80 at E01), but was boosted to ≥ 640 in 51 of 52 participants measured after the first mRNA vaccination (Fig. 4e, median 2,560, IQR 2,250–5,120). No consistent increase in Nt50 occurred after further vaccination. Only participant P845, ambiguous for prior infection, did not reach Nt50 ≥ 640 after vaccination. CD4^+^ T cells provide help to B cells, and S-specific circulating T follicular helper-like cells (cT_FH_) have been associated with disease severity^39^. To determine whether pre-vaccination CD4^+^ breadth might influence post-vaccination antibody neutralization, we conducted a lagged, temporal correlation analysis. The *diagnostic* CD4^+^ breadth metric, shown to be predictive of SARS-CoV-2 infection^22^, at early convalescence (E00) was associated with Nt50 at the same time point (ρ = 0.49, p-value 0.00004) (Fig. 4f). This association of early convalescent *diagnostic* CD4^+^ breadth remained strong with Nt50 after one mRNA dose at E02 (ρ = 0.46, p-value 0.001) (Fig. 4g) but was not present between Nt50 and *diagnostic* CD8^+^ breadth (ρ = 0.2, p-value 0.1). Consistent with this, there was a positive association between Nt50 early after infection and after dose 1 (E02) (Fig. 4h, ρ = 0.38, p-value = 0.007). The rapid increase in Nt50 in persons with hybrid immunity after a single mRNA vaccine dose contrasts with CD8^+^ T cell expansion, which accumulates over both doses 1 and 2.

## Discussion

Given the high global prevalence of hybrid immunity from combined natural infection and vaccination, we sought to understand how sequential exposure to S impacts the circulating TCR repertoire. We phenotyped single cells selected by expression of activation markers upon S peptide stimulation with barcoded mAbs, identifying similar numbers of unique S-reactive CD4^+^ (n = 2430) and CD8^+^ (n = 2467) TCRαβ clonotypes from 24,557 S-reactive single cells. Tracking these clonotypes longitudinally - from convalescence through booster vaccination - in participant-matched bulk TCR repertoires, we observed divergent kinetics between S-reactive CD8^+^ and CD4^+^ T cells. Across almost all participants, comparisons of clonotype frequencies from before and after vaccination were marked by pronounced, vaccine-induced expansions of S-specific CD8^+^ clones: 93% of the highly vaccine-expanded S-reactive clonotypes were CD8^+^ (Fig. 2a, 2b) - with fold-expansions ranging from 4- to > 100-fold in response to mRNA dose 1 and further expansions observed after dose 2.

Despite modest CD4^+^ T cell clonotype expansion after vaccination, we observed that CD4^+^ T cell breadth, measured using *TRB* sequences assigned to S, waned during convalescence but was boosted by vaccination to levels observed soon after acute illness. This may represent detection of both clonotypes recruited from the naive population and proliferation of memory clonotypes. Moreover, in our cohort, we found evidence that severe disease may leave an imprint on the S-reactive CD4^+^ T cell memory subset that persists through vaccination. Participants who were hospitalized had a greater diversity of S-reactive CD4^+^ T cells before and after vaccination. This may reflect the long-lived infection-imprinted IFN-γ and IL-10 cytokine profile of CD4^+^ T cell memory reported by Rodda *et* a/.^15^ in response to natural infection but not vaccination alone. Severe COVID-19 has previously been associated with SARS-CoV-2-specific CD4^+^ T cells with a cytotoxic phenotype soon after recovery^40^ or with an altered Th2/Th17 balance^41^, and with CD8^+^ T cells with markers of exhaustion^42^. It is not yet known if these properties segregate by clonotypes or are imprinted by infection for carryover after vaccination amongst S-specific T cells. In contrast to S-reactive CD4^+^ T cells, neither vaccine-associated nAb titers nor CD8^+^ T cell magnitude or breadth were strongly associated with COVID-19 severity in our study.

Rapid expansion of CD8^+^ T cells has been described in naive persons receiving a first dose of mRNA vaccination^29^; however, continued boosting of S-reactive CD8^+^ T cells by repeated S antigen exposure in COVID-19-recovered persons has not previously been described. Indeed, in naive persons, mRNA vaccines generally induce weaker CD8^+^ T cell responses than replication-incompetent adenovirus vaccines encoding the same antigen^43^. Pseudouridine modification, used in SARS-CoV-2 mRNA vaccines, has been shown to upregulate Th1 recruiting cytokines in mice^44^ and produce strong CD4^+^ Th1 response in humans^45^.

Painter *et* al^46^ compared CD4^+^ and CD8^+^ T cell kinetics in a cohort receiving mRNA vaccination after SARS-CoV-2 infection, measuring the percentage of AIM^+^ blood CD4^+^ or CD8^+^ T cells. They found little CD8^+^ T cell increase after mRNA vaccination, while CD4^+^ T cell boosting was prominent. Several factors may account for the contrast between reports. The peptides used by Painter *et al*. to measure CD8 responses (CD8-E^47^) spanned the SARS-CoV-2 genome, did not include all of S, and contrasted with use of S-covering peptides for CD4^+^ T cells in the same report. As mRNA vaccines encode S, we restricted study to S, and used overlapping 15-mer peptides tiled across the full S protein to include responses to both known and unknown epitopes. The cohort studied by Painter *et al*. was younger and studied sooner after mRNA vaccination than ours, and the intervals between infection and vaccination were not reported. Criteria for assignment of cells as S-specific also varied. CD8^+^ T cell AIM positivity in Painter *et al*. required expression of 4 or more of CD200, CD154, CD137, CD107a, and IFN-γ. Amongst these, CD154 is seldom observed on activated CD8^+^ T cells (as tested herein, **Supplemental Data Figs. 4, 5**, and reported^48,49^), such that congruency for the other markers was likely essential. The markers used for CD4^+^ T cells included CD200, differing from our use of CD69 and CD137. As discussed in Methods, a complex set of activation marker patterns can be expressed by S-reactive PBMC in the hybrid immunity context. Further work will be required to determine if this phenotypic diversity is associated with CD4^+^ T cell clonotype identity, and overall to understand tradeoffs between activation markers for sensitivity, specificity, and capture of T cells with different effector and memory phenotypes and expansion kinetics.

Strengths of this report include the long duration of serial *TRB* repertoire sequencing, up to 2 years, and study of 3–8 blood samples sequenced per participant. This permitted us to measure the frequency of S-reactive clonotypes from several weeks after infection until after three doses of vaccine. Critically, this allowed quantitative assessment of the contribution of post-infection memory to vaccine-elicited responses. We showed that the main contributor to CD8^+^ T cell responses in hybrid immunity is the expansion of memory cells detected in circulation after infection. However, we also observed that the first two doses of vaccine appear to further increase circulating S-reactive clonotypic diversity, consistent with data from peptide-HLA oligomer-sorted T cells^20^. Another strength of our study was the confirmation of many vaccine-expanded TCRs (analyzed in Fig. 1) as functionally S-specific using AIM (Fig. 2) or TCR reconstruction (Fig. 3). The large degree of overlap between expanded and scAIM-TCRseq-confirmed clonotypes indicates that comparison of serial blood *TRB* repertoires may be a suitable surrogate for resource-intensive AIM- or peptide-HLA oligomer-scTCRseq to assign *TRB* sequences as vaccine antigen- specific. AIM-scTCRseq data from this report substantially augment databases^50^ used to match to estimate T cell responses to vaccines, as reported for adenovirus-based SARS-CoV-2 products^51,52^. While limited data suggest that intramuscular mRNA vaccination alone can result in nasal S-specific CD8 + T cells^11^, more research is required to assess the contribution of a priming or breakthrough infection^12^ for the emplacement of SARS-Cov2-specific T_RM_ in the nose. Here we show that vaccine-expanded clonotypes in circulation are detectable in the nose in persons with hybrid immunity. With development of nasally-delivered SARS-CoV-2 vaccination^53^, nasal swabs and immune sequencing become a rational assay endpoint to evaluate mucosal immunity after vaccination. More speculatively, the novel S-specific TCR motifs identified here (Fig. 3, **Extended Data Fig. 8, Supplemental Data Fig. 2**) are candidates for non-exact, TCR distance-informed matching to serial blood or mucosal TCR repertoires from SARS-CoV-2 vaccine recipients. As knowledge of TCR-peptide-HLA triads grows, TCR sequencing-based immune monitoring can be benchmarked against T cell functional assays.

The use of an HLA-peptide oligomer-independent approach to identify and phenotype S-reactive T cells allowed us to identify and quantify immunogen-specific T cells in a relatively unbiased fashion, without foreknowledge of epitopes or restricting HLA. Thus, we were able to recover thousands of paired chain TCRαβ sequences, revealing both α- and β-chain sequence features contributing to epitope specificity. In particular, we report the first large set of public paired receptors sequence motifs, with experimental validation, for an immunodominant A*03:01-restricted S epitope. As is shown here, S specificity can result from TCRαβ pairs where a single alpha chain may pair promiscuously with multiple beta chains, and vice versa, confirming that isolated β (or α) chain sequencing may be imperfect for assigning specificity from single-chain sequencing. Through experimental validation and cloning of representative TCRs within novel clusters of convergent S-reactive receptor sequences, we show a roadmap for de-orphaning TCR-peptide/HLA ligand pairings. This methodology would be valuably transferred to cohorts in less well-studied and geographically diverse populations with a distinct distribution of HLA alleles, which may not otherwise have cellular responses amenable to study by existing HLA-peptide oligomer reagents.

Our study has several limitations. We were only able to study hybrid immunity in the context of infection before vaccination. Given breakthrough infections after vaccination are now common, work is needed to measure how the order of hybrid exposure shapes the TCR repertoire. Our study was limited to persons infected early in the pandemic who were generally older (median age 60.6 years) and may not directly translate to younger persons. Also, while in general we found considerable overlap between *TRB* sequences expanding across serial blood *TRB* comparison and post-vaccine AIM-scTCRseq, many clonotypes that expanded with vaccination did not match a S-reactive receptor from single cell analysis. Finite sampling of AIM-scTCRseq may contribute to partial coverage of low abundance vaccine-expanded clones. Another factor could be our use of CD69/CD137 as activation markers, which would likely impact CD4^+^ clonotype detection more than CD8^+^ T cells. On the other hand, our use of 15 amino acid-long S peptides is more likely to underestimate CD8^+^ than CD4^+^ responses, given the greater permissiveness of HLA class II than HLA class I for binding of peptides with N- or C-terminal extensions^54^. Thus, we hypothesize that many of the strongly expanded clonotypes not matching at *TRB* with cells recovered by AIM-scTCRseq represent CD8^+^ T cells that are sub-optimally presented in our *in vitro* AIM assay. Detailed analyses of defined peptide sets and activation marker combinations may enable optimization in the future. Our study was limited to mRNA vaccination. Keeton *et al*. studied Ad26.COV2.2 vaccination after infection and observed relatively equivalent boosts of S-specific CD8^+^ and CD4^+^ T cells. The assay used, intracellular cytokine staining, is similar to AIM as it is dependent on the phenotypic markers chosen to denote T cell activation^55^. In contrast, clonotype expansion (as in Fig. 1), while not testing T cell functional potential, is independent of prior assumptions about T cell phenotype.

Possibly, some expanded clonotypes may have been coincidentally amplified by non-SARS-CoV-2 antigen exposure over the vaccination time interval. We searched *TRB* from expanded (Fig. 1) and AIM-scTCRseq (Fig. 2, 3) clonotypes for *TRB* assigned to other antigens in public references^50^. Fewer than 0.1% of these TCRs matched EBV or cytomegalovirus (CMV)-assigned CDR3 sequences. In almost all these cases, neither *TRBV* or *TRBJ* matching nor HLA matching between reported HLA restricting alleles and participant haplotypes, could be documented for these CDR3 sequences assigned to common herpesviruses. One TCR assigned to Epstein-Barr virus (EBV) matched a *TRBV, TRBJ*, CDR3, and participant HLA allele to a clonotype significantly expanded after dose 2. This is not unexpected, as EBV-specific T cells have been detected in several pathophysiologic states, though their significance is unknown^56–58^.

Another limitation of our study is the inability to directly infer whether vaccine-expanded clonotypes that were below the limit of detection prior to vaccination came from very rare memory or from naive populations. Conclusive identification would require sorting large numbers of PBMCs from seropositive persons from time points prior to vaccination for separate repertoire sequencing, or identification of naive S-reactive clones by comparing the outcome of vigorous peptide stimulation protocols^42^ between purified naive and memory cells, which was not feasible in our cohort. Despite not being able to definitively resolve whether the vaccine elicits naive cells at the time of vaccination in previously infected persons, we showed unambiguously that the mRNA vaccination expands many low-abundance CD8^+^ clonotypes previously undetectable after infection.

In summary, vaccine formulations of mRNA encoding SARS-CoV-2 S in lipid nanoparticles, administered intramuscularly, induce profound, albeit variable, expansion of pre-existing circulating memory T cell clones. Given the ability of VOC to escape antibody responses, it is likely that virus-specific T cells contribute to protection, at least from severe disease, in the hybrid immunity context. Phenotypic analyses show that vaccine-driven clonotype-level expansion is much greater for CD8^+^ than for CD4^+^ T cells, while the overall diversity of circulating S-reactive CD8^+^ and CD4^+^ T cells after mRNA vaccination is similar. Sequence variation amongst S-specific TCR heterodimers, while large, is amenable to simplification by clustering algorithms. This allows accurate prediction of the HLA restriction of previously unseen TCRs. Further research is required to determine how the phenotype, durability, and distribution of CD8^+^ T cells elicited by hybrid exposures compare to responses elicited by infection or vaccination alone.

## Methods

### Participants and specimens.

Persons with a self-reported history of COVID-19 were recruited during Spring and Summer 2020 in the Seattle area for convalescent plasma donation (NCT 04338360, 04344977). Participants provided informed written consent for University of Washington Institutional Review Board protocol STUDY00004312 “Protocol for the Collection of Laboratory Research Specimens”. PBMC were cryopreserved at 5–10 X 10^6^ cells/vial in 10% DMSO, 50% human serum, 40% RPMI-1640 in LN_2_. Plasma from heparin-anticoagulated blood or serum was frozen at −20°C.

### SARS-CoV-2 antibody assays.

Plasma neutralizing antibodies against SARS-CoV-2 strain WA1 were measured by microfluorescence^23^ and resulted as the reciprocal dilution inhibiting infection by 50%. Plasma SARS-CoV-2 anti-strain Wu-1 S and anti-N IgG were measured by microbead-based binding assay^59^ and reported as μg/ml.

### AIM detection and sorting of SARS-CoV-2-reactive T cells.

PBMC were thawed and cultured at 4 X 10^6^ cells/well in 2 mL/well T cell medium (TCM)^60^ in 2 to 3 wells of a 24-well plates with 1 μg/mL each peptide covering SARS-CoV-2 strain Wu-1 Spike (PM-WCPV-S-1, JPT, Berlin, Germany) in a final concentration of 0.2% DMSO, or DMSO negative control. Peptides were 15 amino acids long with 11 amino acid overlap. After 18 hours, cells were recovered by centrifugation into 50 μL PBS with 1% BSA and incubated with 5 μL TruStainFcX^™^ blocking reagent (BioLegend) for 10 minutes on ice, followed by addition of 50 μL of a cocktail (**Supplemental Data Table 9**) of oligonucleotide-labeled mAbs (Totalseq^™^ C, BioLegend) for 5 minutes on ice. Cells were then stained with anti-CD3-FITC (SK7, BioLegend), anti-CD69-BV421 (FN50, BioLegend), anti-CD137-APC (4B4–1, Becton Dickinson), and 7-actinomycin D (7-AAD, Becton Dickinson), washed and resuspended in 1 ml TCM. Live, single, CD3^+^ cells expressing CD69 and CD137 were sorted (FACSAria II, Becton Dickinson) from S-stimulated PBMC for subsequent AIM-scTCRseq (representative gating tree, **Supplemental Data Fig. 4a**).

To study AIM combinations, PBMC were stimulated and stained with anti-CD3-PE (SK7, BioLegend), anti-CD4-APC-H7 (RPA-T4, Becton Dickinson), anti-CD8-FITC (3B5, Life Technologies), anti-CD25-PE-Cy7 (BC96, BioLegend), anti-CD134-BV480 (L106, Becton Dickinson), anti-CD137-APC (4B4–1, Becton Dickinson), anti-CD154-BV711 (24–31, BioLegend), and 7-AAD. After washing, data was acquired on a FACSAria III in analytic mode. Gating and Boolean analyses used FlowJo 10.7.1 (Becton Dickinson). Gating schemes are shown in **Supplemental Data Fig. 4b**. We investigated activation of CD4^+^ T cells by measuring TNF receptor (TNFR) family members CD134 (OX40L), CD137 (4–1BB), and CD154 (CD40L), and CD25 (IL-2 receptor subunit), and CD69. We considered five pairs of activation markers: CD69/CD137, CD25/CD134, CD134/CD137, CD134/CD154, and CD137/CD154, and set the sum of cells expressing at least two markers to 100%. For CD8^+^ T cells, CD69/CD137 detected a median of 85.7% of all activated cells, with CD134 and CD154 showing very little activation (**Supplemental Data Figs. 4c,d, 5**). For CD4^+^ T cells, the sensitivity to detect peptide activation using CD69/CD137 was more limited (median 21.1 %). However, no AIM molecule pair was consistently the most sensitive. CD69/CD137 showed better sensitivity than combinations of two TNFRs and was used to identify both CD4^+^ and CD8^+^ T cell activation by peptide stimulation.

### Bulk TCR sequencing.

Genomic DNA was extracted from frozen PBMC samples using the Qiagen DNeasy Blood Extraction Kit (Qiagen). Immunosequencing of CDR3 regions of TCR-β chains used the ImmunoSEQ^™^ Assay (Adaptive Biotechnologies, Seattle, WA, USA). Input DNA was amplified in a bias-controlled multiplex PCR, followed by high-throughput sequencing. Sequences were collapsed and filtered to identify and quantitate the absolute abundance of each unique TCR-β CDR3 region for further analysis, as described^35–37^. For analyses of bulk repertoires, the term clonotype is used for a unique *TRB* sequence: a complementarity determining region 3 (CDR3) nucleotide sequence and associated *TRBV* and *TRBJ* genes. These generally distinguish a unique T cell clonotype; occasionally, a single *TRB* may pair with > 1 *TRA* in distinct T cell clonotypes.

### Single cell TCR sequencing.

Sorted single cells were segregated into nanoliter droplets (Chromium Next GEM Single Cell 5’ Kit v2, PN-1000263, Chromium Next GEM Chip K Single Cell Kit, PN-1000286, Chromium Controller, 10X Genomics, Pleasanton, CA, USA). The VDJ and antibody feature barcode libraries were made per user guide (CG000330 Rev C, 10X Genomics). Library quality was measured by TapeStation (Agilent, Santa Clara, CA, USA). Library DNA quantification was measured by Qubit 3.0 Fluorometer (ThermoFisher, Waltham, MA, USA). Sequencing depth was 5,000 paired reads per cell with configuration 26, 10, 10, 150, assuming 10,000 cells per library. Sequencing used the NovaSeq 6000 System (Illumina, San Diego, CA, ISA) and SP200 kit (Illumina).

### Single-cell VDJseq and feature barcode data analysis and alignment.

Raw sequencing data were processed with the Cell Ranger version 6.1.0 (10X Genomics) pipeline. Demultiplexing from raw .bcl data and conversion to .fastq data used Cell Ranger mkfasq. Surface feature barcode antibody binding analyses used Cell Ranger counts with the reference feature barcode library (**Supplemental Data Table 8**). TCR VDJ analyses used the Cell Ranger VDJ module and GRCh-Alts-ensembl-5.0.0. Output matrix data files for feature barcodes and TCR were initially analyzed with the Loupe package (10X Genomics).

### CD4 ^+^ /CD8 ^+^ assignments.

For each cell we computed the percent of UMI counts corresponding to DNA barcodes for CD8 and CD4 assigned to each marker. To assign a phenotype per cell, we computed a score based on the natural logarithm of total CD8 divided total CD4 counts. A score greater than 1 was classified as CD8 and a score less than - 1 was classified as CD4. Values between 1 and - 1 were considered ambiguous and not assigned a T cell phenotype. When a TCR clonotype (cells with identical *TRA* and *TRB* nucleotide sequences) was present in multiple cells, the median score was used to classify that clonotype.

### Longitudinal analysis of S-reactive clones.

We tested for enrichment of S-reactive clones in the AIM assay using a statistical test. The observed frequency of each AIM^+^ clonotype among the total AIM + cells was compared with an expectation from a null model based on each clonotype’s frequency in the bulk sequenced repertoire from the same sample. The p-value of the observed counts of each AIM + clonotype under the null model was computed from the complement of the binomial cumulative distribution function:

$$1-Pr\left(X<k-1\right)=1-\sum_{i=0}^{k-1}\left(\frac{n}{i}\right)p^{i}{\left(1-p\right)}^{n-i}$$

From the binomial cumulative distribution function, we computed the chance of observing *k* single cells of a given clonotype in a pool of *n* total AIM^+^ single cells, with the null success probability *p* equal to the fraction of the matching *TRB* in the unsorted bulk repertoire. We then applied a multiple hypothesis correction with the Benjamini-Hochberg procedure to compute an FDR-adjusted q-value for each AIM^+^ clonotype. We designated clonotypes with a q-value < 0.05 as stringently enriched by the AIM sort and thus high confidence S-reactive clones. These highest-confidence S-reactive clonotypes were used for trajectory analysis and estimation of the total fraction of the repertoire composed of S-reactive CD4^+^ and CD8^+^ T cells, respectively.

### TCR sequence clustering.

To compare and cluster paired *TRA/TRB* sequences between cells, we first filtered sequences from AIM-scTCRseq to those with a matching *TRB* from the deeply sequenced bulk blood repertoire from the same subject and time point. Next, we filtered out sequences with *TRB* occurring at a lower frequency in the set of clonotypes expressing AIM markers than in the bulk repertoire, clonotypes which had not been enriched by AIM. Non-enriched clonotypes were assumed to have been sorted after bystander activation and were not considered further. Next we computed pairwise dissimilarity between 5569 subject-unique *TRA/TRB* clonotypes using the TCRdist distance metric^27^ as implemented using default parameters in *tcrdist3* version 0.2.2^28^. Pruning the pairwise distance matrix to include connections between sequences within 100 TCRdist units, we formed a sequence graph.

When selecting samples to analyze by TCRdist, we restricted analyses to samples to include ≥ 2 persons with prevalent HLA alleles, such that the subcohort studied included persons with HLA-A*02:01 (n = 11), HLA-A*03:01 (n = 7), HLA-A*11:01 (n = 6), HLA-B*07:02 (n = 6), HLA-B*15:01 (n = 4), HLA-B*35:01 (n = 4), HLA-C*07:02 (n = 8), and HLA-C*03:04 (n = 3), HLA-A*24:02 (n = 2) (**Supplemental Data Tables 1, 3, 10**). To examine whether connected components with the graph (i.e., any subgraph where a pair of nodes is connected with each other via an edge path) might recognize an HLA-restricted epitope, we used a graph walking approach to discover minimal sets of feasible HLA alleles that participants shared within closely connected nodes. Briefly, for each node, we sorted nodes in ascending order by TCRdist to all other nodes with its largest connected component. Starting at the closest public node found in another HLA-genotyped participant, we took the intersection of the set of all class I (CD8^+^ nodes) or class II (CD4^+^ nodes), before moving on to the next-nearest connected node and taking the next stepwise intersection. If possible, the algorithm continues to narrow the set of feasible presenting HLA alleles to a minimal possible set. We inferred feasible HLA alleles, and in many cases only one allele was shared among closely connected S-reactive TCR sequences. This allele was assigned to the corresponding TCR cluster. Code to assign feasible HLA restriction from TCR sequence similarity analyses/graphs is provided in the Code availability section using custom scripts run in Python version 3.9. Graph visualization used the Networkx v2.8.6 package^61^.

### TCR motif visualization.

From the weighted sequence similarity graphs formed from all AIM-scTCRseq S-reactive clones, we identified clusters of similar sequences using the Louvain community detection algorithm with the communities v3.0.0 package in Python. For each public sequence cluster with sequences donated from 3 or more participants, we depict selected TCR clusters by six graphical elements, with the CDR3α and β junctions on the left and right, respectively (Fig. 3b–h). The lower sequence logo shows the observed position-specific frequency of each amino acid within the TCR cluster, and the upper logo plots represent the position-specific information content in bits (i.e., a signal of selection) compared to CDR3α and β receptors, with the same *V-* and *J*-gene usages, randomly sampled from naive repertoires. The Sankey flow diagrams left of the CDR3 motifs show the frequencies of *TRAV/TRAJ* and *TRBV/TRJV* gene usages within each cluster. Motifs were aligned and computed in palmotif v0.4, and graphics rendered using ggplot2 and ggseqlogo^62^ in R version 4.2.

### TRB repertoire analyses.

Breadth of *TRB* sequences significantly expanding (or contracting) between serially collected blood specimens was calculated as the number of unique clonotypes meeting significance criteria. In brief, to determine longitudinal persistence and previous detection, *TRB* were filtered for productive sequences and analyzed at the nucleotide level (*CDR3, TRBV, TRBJ)*. *TRB* from bulk sequencing data were defined as expanded if their log_2_ fold change was > 2 relative to the E01 time point and met a second criteria for a statistically significant change in counts between samples using Fisher’s Exact Test with correction for multiple hypotheses (FDR-adjusted q-value < 0.05). Analysis used custom Python scripts detailed in Code Availability Statement. CDR3 amino acid sequence with V and J gene usage and HLA restriction (if published) was used to determine whether a clonotype had been shown previously to be associated with a known antigen.

Separately, *TRB* CDR3 assigned to SARS-CoV-2 were generated by statistically comparing *TRB* CDR3 sequences from whole blood Immunoseq *TRB* repertoires between persons with documented SARS-CoV-2 infection and healthy controls (HD). Sequences were assigned as likely to represent CD4^+^ T cells based on publicity between persons sharing HLA class II alleles, or as likely to represent CD8^+^ T cells based on publicity between persons sharing HLA class I alleles. Assignments to SARS-CoV-2 S or non-Spike specificity were performed using the output of multiplexed antigen restimulation assays (MIRA)^63–65^. Briefly, defined SARS-CoV-2 antigens were used to stimulate expanded PBMC from SARS-CoV-2-infected persons and sorted CD4^+^ or CD8^+^ T cells expressing activation markers were bulk-sequenced at the *TRB* locus. Further refinements were performed to exclude non-SARS-CoV-2-specific *TRB* sequences associated with ubiquitous antigens such as CMV or EBV, or with *TRB* sequences non-specifically associated with HLA alleles in a cohort of healthy controls^66^. MIRA-enriched and statistically SARS-CoV-2-associated *TRB* sequences were co-analyzed to create sets of *TRB* sequences spanning CDR3 and assigned, when possible, as CD4^+^ or CD8^+^, or as S- or non-S-specific. The *diagnostic* breadth of blood *TRB* repertoires were calculated as described^66,67^ and represent the proportion of productive *TRB* clonotypes present in a repertoire that are assigned as SARS-CoV-2-specific.

### TCR expression.

TCR CDR3 sequences from AIM-scTCRseq were integrated into assigned *TRA* and *TRB* genomic variable genes using IMGT^68^. Codon-optimized TCR lentiviral expression constructs (Genscript, Piscataway, NJ, USA) were cloned into pRRLSIN.cPPT.MSCV/GFPWPRE^69^. The TRB polypeptide is N-terminal within a fusion protein separated from TRA by a porcine echovirus P2A sequence, with both TCR constant regions of murine origin and extra cysteine residues to promote pairing^70^. Lentiviral particles were expressed in HEK293 cells (CRL-1573, ATCC, Manassas, VA, USA) by co-transfection with packaging plasmids^71^. Lentivirus was concentrated (Lenti-X, Takara, San Jose, CA, USA). The NR4A1 mNeonGreen TCR reporter cell line has been described^72^. In brief, mNeonGreen was integrated in-frame into the *NR4A1* locus using CRISPR-induced homology-directed repair into Jurkat E6–1 cells (ATCC). The cells were additionally modified to knock out endogenous TCR expression. CD8αβ were also inserted using CRISPR to create NR4A1_mNeonGreen_035 (Jurkat_035). Jurkat_035 were maintained in TCM. 1 X 10^6^ Jurkat_035 cells were transduced with 200–300 μl of lentiviral stock based on analysis for gag protein (Lenti-X Go Stix, Clontech) for an estimated 5 infectious units per cell. Five days or more after transduction, flow cytometry routinely showed > 80% TCR expression as measured with anti-murine TRB clone H57–597-APC (eBioscience, San Diego, CA, USA).

### Determination of TCR specificity and HLA restriction.

To evaluate specificity, Cos-7 cells were transfected as described^73^ in 96-well flat-bottom plates with HLA class I cDNA, SARS-CoV-2 full length S from strain Wu-1 cloned into pDEST103, HLA and S, or neither. Details of HLA cDNA and S cloning are published^23,71,73–75^. HLA and S were sequence-confirmed. At 48 hours, TCR-expressing Jurkat_035 were stained with Cell Trace Violet (Invitrogen by Thermo Fisher), washed, and added to Cos-7 cells at 3 X 10^5^/well. To begin to determine peptide epitopes TCR-expressing Jurkat_035 were co-cultured with B-LCL (1 X 10^5^ each) and pools of 1 Mg/ml each 15 amino acid peptide covering the N or C terminal halves of SARS-CoV-2 Wu-1 S (PM-WCPV-S-1, JPT, Berlin, Germany) in 200 μL TCM in round-bottom plates. Next, HLA-transfected Cos-7 aAPC and 2 X 10^5^/well CTV-labeled Jurkat_035 were incubated with S peptides, 13 AA long with 9 AA overlap covering full-length SARS-CoV-2 strain Wu-1, arrayed in rectangular matrices with row or column pool complexities of 8 to 12 peptides as reported^23^. Peptides at the intersection of positive rows and columns, or internal shorter peptides, were repeated in follow-up experiments. Subsequent experiments used fresh synthesis of selected single peptides and truncated internal peptides in the vicinity of active peptides, at 1 μg/mL (Genscript, 70% purity). For some peptide experiments, the APC used were autologous EBV-transformed B-lymphoblastoid cell lines (B-LCL). These were cultured as described^76^ and CTV-labeled, in which case Jurkat_035 responder wells were not CTV-labeled. To study S variants, Cos-7 APC were transfected with S constructs representing Wu-1 or SARS-CoV-2 Omicron variants BA.1, BA.2, and BA.4, each with 21 amino acid C-terminal deletions. Regardless of APC and antigen, after 24 hours, cells were analyzed by flow cytometry (LSR II, Becton Dickinson) and the percent of gated Jurkat cells, either CTV-high or CTV-low, expressing mNeonGreen was analyzed.

### HLA typing.

HLA class I and II allotypes were determined by next-generation sequencing at Scisco (Seattle, WA, USA)^25^.

### Statistical Testing.

Statistical comparison between paired samples are signed-rank tests, and comparison between groups are Wilcoxon tests. Associations among immunological parameters were measured with Spearman rank correlation. Asterisks represent level of statistical significance (ns = not significant, *<0.05, **<0.01, ***<0.001, ****<0.0001). Statistical testing was performed in R version 4.0.5.

## Data Availability

PBMC and nasal swab *TRB* repertoires are at ImmuneAccess https://clients.adaptivebiotech.com/immuneaccess. E00 and E00.5 timepoint data are at https://doi.org/10.21417/RE2022JCI. Remove the first two digits of participant IDs to match IDs in this report. Later time point data are in ImmuneAccess with username koelle-review@adaptivebiotech.com and password koelle2022review. Processed AIM-scTCRseq and feature barcode data are at https://zenodo.org/record/6909380#.Yvqcg3bMKUk. Sequences of TCRs expressed in reporter cells are in Genbank as OP245920-OP245935.
